# From Protein Features to Sensing Surfaces

**DOI:** 10.3390/s18041204

**Published:** 2018-04-15

**Authors:** Greta Faccio

**Affiliations:** Independent Scientist, St. Gallen 9000, Switzerland; greta.faccio@gmail.com; Tel.: +417-6210-6221

**Keywords:** surface functionalization, biosensor functionalization, protein immobilization, protein structure analysis, protein immobilization

## Abstract

Proteins play a major role in biosensors in which they provide catalytic activity and specificity in molecular recognition. However, the immobilization process is far from straightforward as it often affects the protein functionality. Extensive interaction of the protein with the surface or significant surface crowding can lead to changes in the mobility and conformation of the protein structure. This review will provide insights as to how an analysis of the physico-chemical features of the protein surface before the immobilization process can help to identify the optimal immobilization approach. Such an analysis can help to preserve the functionality of the protein when on a biosensor surface.

## 1. Introduction

Proteins provide specific recognition for the analyte in biosensors and their immobilization is a crucial component: it can highly affect the performance of the device if electron transfer is not guaranteed or if the protein undergoes major conformational changes that alter its functionality. As compared to small molecules that offer few chemical groups of clear position and solvent accessibility (e.g., dyes in solar cells [1], DNA [2], or aptamers [3]), protein sizes can reach the tens of nanometers and achieve complex three-dimensional structures that dynamically move during bioactivity, with environmental conditions, and especially after coming into contact with material surfaces.

To achieve optimal immobilization in a biosensor can be a complex task, one which often proceeds through trial and error to retain most of the affinity for either the analyte or, in the case of enzymes, enzymatic activity. Immobilization can alter the enantioselectivity of enzymes, as reported for both lipase and acylase which undergo extensive conformational changes during catalysis [4]. Immobilization in a preferred orientation can guarantee the maximal exposure of biorecognition moieties, e.g., catalytic sites of enzymes and antigen-binding sites of antibodies [5], while the protein region interacting with the surface is minimized and limited to regions of the molecule that do not undergo conformational changes during biorecognition. Oriented immobilization of enzymes has been proven to lead to a higher catalytic activity on the surface as compared to random immobilization [6]. These are critical aspects when working with enzymes for industrial biocatalytic applications, as recently reviewed [7].

Significant loss of function often results from extensive conformational changes and partial denaturation of proteins adsorbed or chemically crosslinked to surfaces [8,9]. However, the immobilization process, if well planned, can enhance bioactivity and stability, e.g., a 60,000-fold increase in stability has been reported for chymotrypsin on aldehyde–agarose gels [4]. By carefully selecting the material, its coating, and by studying the properties of the protein to immobilize, it is possible to tune their interaction through single or multiple points, using flexible or rigid linkers, in hydrophilic or hydrophobic environments to protect the protein and to prolong its functionality through multiple cycles of use [4]. This review will provide an insight into different immobilization approaches and how the study of the protein structural and surface features can help to identify the optimal immobilization approach to ensure the retention of the highest degree of functionality once assembled in the biosensing device. An overview of the developed strategies of proteins immobilized and their outcome are also discussed.

## 2. Protein Surface and Function

**Figure 1:**
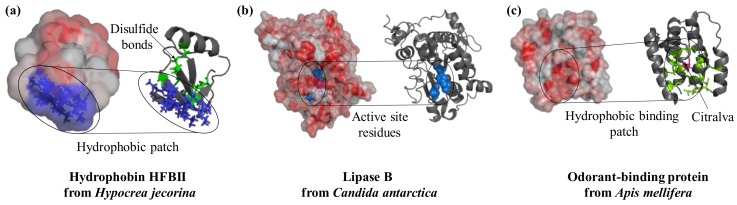
Surface features play a crucial role in the function of proteins. Hydrophobins interact with substrates in a specific region of the structure that is rich in hydrophobic residues (PDB ID: 2b97, blue). Lipases are active enzymes on hydrophobic substrates; they catalyze the hydrolysis of triacylglycerides and their active site (key residues in blue and spheres) is located in a hydrophobic protein patch (PDB ID: 4k6g). Similarly, the odorant-binding protein from bees has a hydrophobic cleft (residues in green as sticks) at the center of the molecule to bind the perfume-like water-insoluble molecule citralva (in pink sticks, PDB ID: 3s0d). Protein structures are visualized with Pymol (The PyMOL Molecular Graphics System, Version 1.2r3pre, Schrödinger, LLC.), surface hydrophobicity was analysed by running the script “color_h” [19] and surface electrostatic potential with the APBS plugin [20].

Proteins differ widely in their biological functions and this is reflected in specific structural features [10]. Proteins are surface-active molecules and the distribution of charged and hydrophobic residues on their surface is often the basis of their functionality (Figure 1). For example, hydrophobins are characterized by a well-defined hydrophobic patch on their surface which drives their interaction with surfaces and interfaces in a highly oriented manner (Figure 1a). Hydrophobin from *Schizophyllum commune* has been used to alter the properties of glassy carbon electrodes in a single self-assembly step prior to the immobilization of redox-active glucose oxidase and horseradish peroxidase [11]. This process led to an adsorbed multi-layer assembly of glucose oxidase with thicknesses of 79 Å and 173 Å for horseradish peroxidase, which were both permeable to the analytes and allowed an efficient electron transfer [11]. Enzymes such as lipase (Figure 1b) and cholesterol oxidase, which are active on hydrophobic substrates, often present an enrichment of hydrophobic residues in the proximity of the active site. Lipase B from *Candida antarctica* is strongly adsorbed to hydrophobic surfaces such as graphite [12] and porous styrene–divinylbenzene beads [13]. Immobilization of lipase from *Pseudomonas cepacia* into siliceous mesocellular foams with different degrees of hydrophobicity demonstrated how increased hydrophobicity led to an enhancement of catalytic activity [14]. Enzymatic activation was a result of an interaction with the material; this led to an opening of the hydrophobic lid covering the active site in many lipases [7,14]. Similarly, odorant-binding proteins (Figure 1c) are small 13–16 kDa proteins naturally secreted in vertebrate nasal cells to bind hydrophobic odorant molecules. These proteins have proven valuable in the development of bioelectronic noses and odor biosensors. Immobilization of these proteins to nanomaterials has reached detection limits of 0.02 ppt molecules [15] and function in both gas and liquid phase [16]. The crystal structure of protein 14 from *Apis mellifera* is available and show six α-helices whose hydrophobic residues form a hydrophobic core that binds the odorant molecule [17]. Immobilized to reduced graphene oxide with a short 1-pyrenebutanoic acid succinimidyl ester (PBSE) linker, protein 14 retained affinity for the aromatic molecules homovanillic acid, eugenol, and methyl vanillate with *K*_d_ values in the micromolar range; however, the binding provoked a slight reorientation of the α-helices [18].

## 3. Protein Structure, Surface and Material Surfaces

Based on their structural stability, proteins have long been classified as ‘soft’ or ‘hard’ proteins according to their structural flexibility or rigidity, respectively [10]. Whereas soft proteins are characterized by a high flexibility and are less thermodynamically stable, hard proteins are less structurally affected by high temperatures, environmental conditions, and their conformation is mainly conserved upon interaction with material surfaces. The application of a difference of potential to the electrode can affect the behavior of the proteins at the surface; as the protein structure contained dipoles and charged residues, this enhanced the degree of adsorption of proteins, especially those classified as hard [21]. Examples of soft proteins are myoglobin, α-lactalbumin, glucose oxidase, immunoglobulin G, and caseins; in contrast, hard proteins are often characterized by multiple disulfide bonds that help to counteract the denaturation such as in lysozyme, ribonuclease A, and acetylcholinesterase. Bovine serum albumin (BSA), which possesses seventeen disulfide bonds [22], is a hard protein and one of the most frequently used model molecules to test the interaction of a material with proteins and to mimic its behavior in physiological fluids. The use of model proteins such as BSA is convenient but has limitations as the information cannot be directly applied to any other protein for use in surface functionalization, such as glucose oxidase for blood glucose monitoring or antibodies for biomarker detection.

Tightly interacting secondary structure elements and disulfide bonds confer molecular rigidity and help to preserve overall conformation, whereas hydrophobic or densely charged surface patches can drive the interaction with specific surfaces. The surface of a protein can present a highly heterogeneous distribution of charges and hydrophobicity which may influence its solubility, stability, and functionality in different environments [23,24,25,26]. Accordingly, protein surface features play a crucial role in the conformational stability of proteins. Moreover, such features control the interaction of proteins not only with material surfaces but also with other biomolecules which can modify their behavior as well as stabilize or destabilize their structure and compromise their bioactivity [27,28,29]. In addition, surface topography, e.g., the roughness or curvature in the 15−165 nm range, has been reported to alter secondary structure elements in a protein-specific manner [30,31,32]. To understand and control this interaction, the in vitro and *in silico* analysis of protein surfaces is a crucial step for future engineering efforts. For example, the green fluorescent protein (GFP) could be selectively adsorbed to the positively charged regions of a patterned coated surface after analysis of its surface features [33].

**Figure 2:**
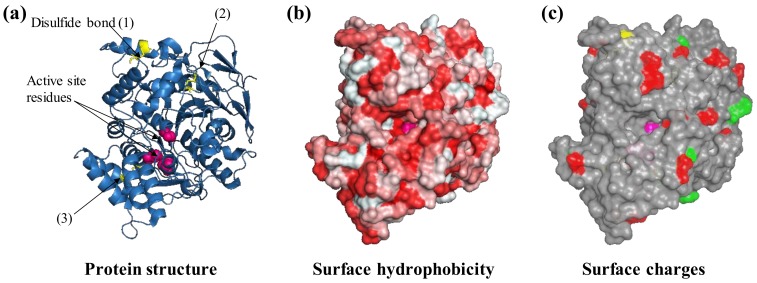
Protein features to consider before selecting an immobilization strategy. For example, the protein acetylcholinesterase from the electric eel *Electrophorus electricus* (PDB ID: 1c2b) is shown. The three-dimensional structure of acetylcholinesterase is shown as ribbons (**a**) with three visible disulfide bonds (yellow sticks) and active site residues highlighted (pink spheres, **b**), negatively (green) and positively charged Lys residues (red) that are exposed on the surface (**c**). Protein structures are visualized with Pymol (The PyMOL Molecular Graphics System, Version 1.2r3pre, Schrödinger, LLC.).

Among other forces, adsorption of proteins to surfaces is driven by hydrophobicity and ionic or electrostatic interactions. When electrostatic attraction is the driving force, the adsorption process is highly influenced by environmental conditions such as pH and ionic strength as the ions in solution can shield the charges on the surface of both the protein and the surface. For example, proteins with a net positive charge, which have a surface rich in arginine residues but poor in aromatic ones and are characterized by a low structural rigidity, are more prone to adsorption to the negatively charged hydroxyl-rich surface of biosilica [34,35]. Although the analysis of a protein’s structural features may be complex, it can offer hints as to the optimal immobilization strategy. Surface hydrophobic patches of soluble proteins are not only rich in Ala, Lys, and Pro residues but also can have areas of 400 Å; they often drive multimerization or undesired aggregation as well as interactions with hydrophobic materials, e.g., those found in cellulose-active enzymes and lignin [36]. Using acetylcholinesterase (AchE) as a model, we can observe the key structural elements which can affect the behavior of a protein with a surface (Figure 2).

AchE is an enzyme naturally involved in synaptic signal transduction in which it hydrolyses acetylcholine to choline and acetate. It is also a widely used enzyme in biosensors as a result of its sensitivity towards pesticides and pharmacological molecules that are utilized in the treatment of neurological disorders, e.g., Alzheimer’s disease [37]. AchE is also a biocomponent in biosensors for the detection of aflatoxin B1 and organophosphate poisons in general [38,39]. The AchE molecule is characterized by four disulfide bonds and can thus be considered a relatively hard protein. The protein surface presents a certain degree of hydrophobicity that can drive the interaction with hydrophobic substrates such as graphene oxide as well as modified hydrophobic gold nanoparticles [40,41] (Figure 2b). In solution, the enzymes can undergo dynamic multimerization [42]. The immobilization of acetylcholinesterase to a modified hydrophobic surface has been reported to not only not cause denaturation and loss of functionality [41] but also result in more than 1000-fold enhancement in the affinity for toxic organophosphor compounds and in a 110% increase in thermal protein stability [43].

The surface of AchE presents residues carrying carboxylic (Asp, Glu) and amine groups (Lys, Gln, Figure 2c) that can be used for protein immobilization by chemical means or drive adsorption. As a bifunctional chemical crosslinker, glutaraldehyde can be used to covalently bind proteins such as protein A through these residues to later immobilize antibodies by affinity to amino-decorated surfaces, e.g., coated with polyethylenimine or polylysine [44]. AchE amine groups have also been used for immobilization by EDC/NHS chemistry to a carboxylate-modified silicon substrate [45] to detect organophosphorous pesticides [45]. Immobilization of AchE has been performed to modified carbon electrodes carrying dialdehyde moieties (covalent immobilization) or after coating with polyethyleneimine (physisorption); both approaches resulted in a reduction of the affinity for the analyte, i.e., an increase in *K*_m_ [46]. In an alternative approach, the entrapment of AchE in the hydrophilic polymer chitosan protected the enzymatic activity and provided functionality in the presence of methanol (25%), acetonitrile (15%), and cyclohexane (100%) conditions in which an equivalent preparation from chemical crosslinking with glutaraldehyde lost activity at a much lower concentration of organic solvents [47].

Surface-exposed lysines are residues often used for fluorescent labelling of the protein for easier tracking; however, the behavior of proteins whose surface has been modified with covalently bonded fluorescent dyes can be quite different from native ones [48]. Modification of protein surfaces is possible and can significantly tune bioactivity [49,50], control adsorption to material surfaces and interfaces [51], suggest immobilization strategies to enhance enzymatic activity [52], allow specific protein labelling [53], interact with smaller biomolecules such as peptides [54], and be used for molecular detection [55]. The tuning of the degree of a protein’s surface hydrophobicity is possible. Reduction of surface hydrophobicity of AchE by individually substituting 14 solvent-exposed hydrophobic residues with arginine often resulted in an increased stability to temperature and chemical denaturation [56]. In an opposite strategy for the lipase from *Pseudomonas* sp., the introduction of hydrophobic surface patches by site-directed mutagenesis resulted in an increased stability in organic solvents [57]. Glucose oxidase, especially from *Aspergillus niger*, is widely used in biosensors for glucose monitoring and it has a dimeric 160 kDa structure whose units are held together by hydrophobic and hydrophilic interactions, e.g., salt bridges and hydrogen bonds. Covalent bonds such as disulfide bonds are not the only contributors to protein stabilization as the introduction of multiple weak interactions, such as salt bridges on the surface of the proteins, to counteract thermal denaturation has been well established. The modification of the surface of glucose oxidase to carry both a novel sulfur–pi interaction and a salt bridge led to a three-fold increase in thermal stability [58]. These surface substitutions did not affect the glycosylation pattern of the enzyme which was also reported to enhance the thermal stability through the introduction of structural rigidity [59]. Accordingly, its covalent immobilization by entrapment into gelatin using 1-ethyl-3-(3-dimethylamino-propyl)carbodiimide (EDC) increased the melting temperature from 58 °C to 76 °C [59]. Notably, these substitutions affected not only the stability but also the catalytic activity.

The behavior of a protein towards charged surfaces can be estimated through an analysis of its surface and surface charge distribution [47] (e.g., widespread or localized) by calculating its net charge *in silico* [60] or by experimentally determining its surface zeta potential under different pH conditions [61]. Using *in silico* simulations, one can even predict the orientation of the protein on a surface by evaluating the possible protein-surface electrostatic interactions [62]. Proteins are prone to aggregation when environmental conditions are close to their isoelectric point and they also tend to adsorb to surfaces in higher amounts under these conditions [63]. To achieve immobilization based on electrostatic forces and charged amino acids, the addition of negatively or positively charged stretches of amino acids to one terminus of the protein might be a valuable yet reversible approach [64,65]. A positively charged polyarginine tag was attached to the green fluorescent protein that could be immobilized reversibly to mica, glass, and silica surfaces with no loss of activity [64,66]. 

## 4. Protein Immobilization Approaches

**Figure 3:**
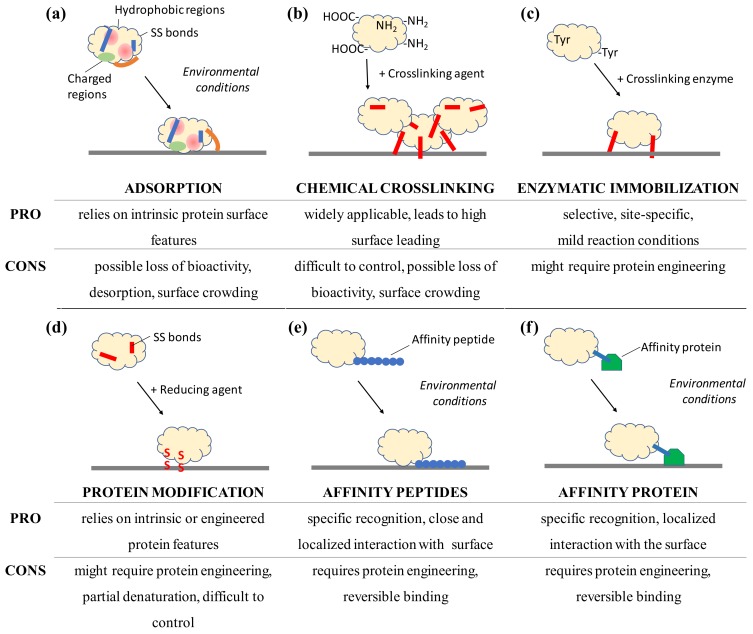
Schematic view of selected immobilization strategies for proteins to surfaces and the advantages and disadvantages to consider after analyzing the protein structure and its surface features. Immobilization by adsorption and affinity rely on environmental conditions, not on the presence of a catalyst or reactive molecule, such as an enzyme, reducing agents or chemical crosslinkers that introduce covalent bonds (in red). Adsorption can lead to conformational changes such as the opening of the hydrophobic lid (orange) which covers the active site of lipases.

A thorough *in silico* and experimental study of the surface protein features can indicate the presence of exposed disulfide bonds or residues susceptible to immobilization by chemical enzymatic crosslinking. Proteins tend to interact with surfaces spontaneously. To preserve protein integrity, immobilization in a site-specific and oriented manner is often desired and has been proven to produce surfaces with a high catalytic activity and a high binding capacity with enzymes, affinity proteins, and antibodies [6,67,68]. Site-specific immobilization involving residues from well-structured portions of the protein molecule has been reported to ensure a higher retention of activity in enzymes. In contrast, immobilization through residues in flexible regions might allow a higher interaction of protein residues with the surface, leading to denaturation [69]. Sites for site-specific immobilization and oriented immobilization can be achieved by using protein engineering to introduce affinity peptides or specific residues, e.g., unnatural amino acids or cysteines, in selected locations or by detailed analysis of the surface properties of the molecule, e.g., charge distribution in antibodies [70]. Deposition as a single monolayer is also beneficial to prevent possible inactivation due to surface crowding; protein engineering offers additional possibilities to introduce desired functionalities, e.g., natural and unnatural amino acids, disulfide bonds or affinity motifs, into selected locations of the protein structure. An overview of selected immobilization approaches as well as their advantages and disadvantages can be found in Figure 3.

Adsorption of proteins to surfaces is a spontaneous process, dependent on the intrinsic features of the protein (Figure 3a). Various features of the protein influence adsorption, which remains a reversible dynamic process. Adsorption provides a direct single-step approach to surface functionalization. A more stable immobilization has been detected for soft proteins, as hard ones have been shown to bind more loosely to the surface and desorb more promptly, as shown from the comparison of variants of human carbonic anhydrase II, albumin, and α-synuclein [8,71]. Measurement or calculation of the protein isoelectric point can help to identify incubation conditions of a net opposite charge between surface and protein; additionally, *in silico* analysis of the three-dimensional structure, when available, can reveal the distribution of localized patches of specific charged or hydrophobic residues. Adsorption often leads to a multilayer assembly on the surface which results in crowding and changes in protein conformation; this may contribute to a loss of bioactivity [30]. Surfaces characterized by significant roughness [72], hydrophobicity (surface tension) [73,74], and polarity [71] are good candidates for functionalization by adsorption, especially with soft proteins. Once surface regeneration is desired, desorption can be promoted by extensive rinsing with solutions characterized by a high ionic strength solutions, pH conditions under which protein and surface carry the same net charge, high temperatures, and the use of anionic and ionic detergents [75,76,77].

However, adsorption is a reversible phenomenon; a more stable solution is provided by chemical crosslinkers which can give multi-point bonding with the surface and thereby introduce an additional degree of rigidity to the protein. Crosslinking by chemical agents (Figure 3b) relies on the presence of functional groups on the surface of proteins, such as widespread amine groups of lysines and carboxylic groups of glutamate and aspartate residues (Figure 2c). Often used to link proteins to modified surfaces, glutaraldehyde introduces covalent linkages between amine groups and is also used to produce crosslinked protein aggregates in solution. The stabilizing effect detected when proteins are chemically immobilized can be ascribed to the molecular rigidity introduced by the formation of multiple bonds between the protein and the surface. This effect can also be ascribed to newly introduced intramolecular bonds in the proteins, especially at low concentrations of crosslinker [78]. For example, lactate oxidase was immobilized by a chemical crosslinking with EDC; this has proved to be a more stable arrangement, not only in terms of enzyme retention at the surface and improved affinity for the analyte with detection limits in the micromolar range but also by increasing the thermal/operational stability of the biosensor, as compared to the only adsorbed enzyme [79]. I refer the readers to a recent review concerning antibodies [80]. Chemical crosslinking can be considered a widely applicable approach, as protein surfaces often offer functional groups that can be exploited for immobilization, e.g., carboxylic, amine, and thiol groups; additionally, such surfaces carry the compatible chemical functionalities for anchoring. However, optimization of reaction time as well as a concentration of reagent and protein are required steps when bioactivity needs to be preserved to the maximum extent. Similar to adsorption, chemical crosslinking does not guarantee control over the orientation of the protein at the surface and might result in surface crowding. Surface regeneration by protein removal is not possible.

As opposed to chemical crosslinkers, protein engineering and enzymatic bioconjugation might offer site-specific approaches to the functionalization of surfaces with proteins [81]. Enzymatic immobilization approaches (Figure 3c) are also available and offer a high specificity resulting in site-directed immobilization, the need for small amounts of catalysts, and environmentally friendly reaction conditions [81]. The crosslinking enzyme sortase has been used for both protein conjugation and protein immobilization, as it only requires an amino/lysine containing receiving surface following the introduction of the pentapeptide Leu-Pro-Glu-Thr-Gly (sortase tag) by genetic engineering of the protein to be immobilized [82,83,84]. This strategy was followed using the *Staphylococcus aureus* sortase A, in which a fibronectin-binding protein was selectively and site specifically immobilized to sensor chips [85]. Membrane-bound glycosyltransferases were covalently immobilized to an amino-modified sepharose resin [84] and a single-chain antibody to a flow cell biosensor conferred specificity for the cancer biomarker extracellular domain of the epidermal growth factor receptor [83]. Whereas the engineering of a pentapeptide might seem to be an alteration affecting the functionality of the protein, these studies demonstrate how its addition in terminal position to the primary structure did not compromise the bioactivity of the enzymes or antibodies. An alternative approach uses enzymes such as tyrosinase and transglutaminase which recognize single residues and specifically attack surface-exposed tyrosines and lysines, respectively [81]. Tyrosinase has been used for the covalent immobilization of fluorescent proteins and protein A carrying surface-exposed tyrosines to amino-modified surfaces for subsequent antibody capture [86,87,88], i.e., surfaces treated with polyallylamine. Transglutaminase has been used to immobilize alkaline phosphatase engineered to carry a structurally exposed lysine within the tag Met-Lys-His-Lys-Gly-Ser to a glutamine-containing casein layer deposited on a polystyrene surface [89] and to agarose gel beads [90]. Crosslinking enzymes can also be used to achieve in situ entrapment of the proteins. Without genetic engineering, glucose oxidase and lactate oxidase were entrapped using translgutaminase into a network of lysine/glutamine-rich proteins and peptides, e.g., polylysine, polyglutamine, and fibrinogen, which had been produced directly on the electrode surface [91]. These enzymatically prepared electrodes retained more than double the sensitivity upon immobilization and a more than two-fold increase in stability as compared to the glutaraldehyde-prepared ones [91]. The polymerization of L-DOPA by tyrosinase has also been used to synthesize a melanin-like polymeric matrix for the entrapment of glucose oxidase and tyrosinase for amperometric biosensing, reaching a 10 nM detection limit for phenol [92,93]. Enzymatic protein immobilization strategies are highly specific and can be applied with proteins that present surface-exposed target residues. Residues to be recognized by the enzyme should be extremely exposed and in highly flexible regions of the protein, e.g., in the terminal flexible regions. A study of the protein structure focusing on its non-crystallized flexible portions may reveal susceptible residues for enzymatic immobilization. Protein engineering is a powerful tool to add the desired amino acids to the protein of interest, e.g., tyrosine tags, sortase tags, and polylysine tags [84,87,90]. Enzymatic immobilization offers a stable covalent immobilization in a site-specific manner but only for surfaces carrying compatible chemical groups.

It is often desired to achieve a controlled immobilization which provides a low surface crowding (optimally a monolayer) and an optimal orientation of the protein; this is often achieved in a site-specific immobilization. Proteins naturally offer features that can be used for this; however, protein engineering is a powerful tool [94]. Although generally considered to be a lengthy process, a first step to protein engineering might provide advantages such as a single-step process and a minimization of the amount of protein needed to provide an ideal protein monolayer; such features might benefit a later deposition to the material. For example, protein engineering may make it possible to introduce selected chemical moieties at a specific location in the protein molecule by using unnatural amino acids without compromising the bioactivity. More than 50 unnatural amino acids containing thiol, azide, or keto groups [95,96,97] have been incorporated into proteins and the reactivity of the unnatural amino acid with the surface could be used to achieve site-specific immobilization [98,99]. Proteins carrying superficial cysteines (Figure 3d) can be immobilized directly to disulfide-containing materials or gold electrodes [100]. Protein disulfide bonds are chemically reduced to form reactive thiols, as in the case of antibody immobilization through the use of dithiothreithiol (DTT), dithiobutylamine (DTBA), tris(2-carboxyethyl)phosphine (TCEP), or 2-mercaptoethylamine (2-MEA) [80,101]. An alternative UV-light-based technique, which relies on the presence of aromatic residues in the proximity of disulfide bonds, has also been developed to preserve the structure and functionality of the protein while allowing site-specific and space-resolved immobilization. This technique has been applied to a wide range of proteins, e.g., hydrolytic enzymes, proteases (human plasminogen), alkaline phosphatase, antibody against prostate specific antigen major histocompatibility complex class I protein, pepsin, and trypsin [102].

Peptides might be considered simpler structures than proteins because of their smaller size; however, they can be valuable in biosensors and for protein immobilization (Figure 3e). The possibility of synthesizing and designing peptides allows the insertion of desired chemical groups into specific positions, thus introducing functionality. For example, a kinase biosensor has been assembled using a peptide labelled with a fluorescent tetramethylrhodamine (TAMRA) group, i.e., sequence TAMRA-Leu-Arg-Arg-Ala-Ser-Leu-Gly, that produces a FRET signal via a Zn^2+^-coordination with the COOH-rich surface of quantum dots only when phosphorylated by kinases [103]. Peptides can also be fused to proteins at the gene level and used to confer novel affinity features and an oriented immobilization. The oriented immobilization achieved with affinity peptides has resulted in a higher degree of retained catalytic activity when using the His-tag with sulfotransferases [6]. Affinity peptides of different lengths are available for a wide variety of substrates from polystyrene [104] to gold [105], from crystalline sapphire [106] to crystalline nanocellulose [107], and from carbon nanotubes [108] to graphite [109]. Our group engineered a bacterial laccase from *Bacillus pumilus* to carry a terminal affinity peptide for iron oxide; this led to higher protein loading on the surface and a doubling of the enzymatic turnover *k*_cat_, especially when in a monolayer assembly at the surface [110]. Similarly, carbonic anhydrase has been engineered to carry a single-walled-carbon-nanotube affinity peptide that provided not only binding but also a 51% surface coverage while retaining the protein’s secondary structure elements and enzymatic activity [111]. Fused to carry multiple gold-binding peptides, alkaline phosphatase was immobilized to a gold-patterned substrate, giving a higher enzymatic activity per area as compared with the unmodified enzyme [112]. By engineering affinity motifs into the protein molecule and ensuring their exposure on the surface of the molecules, a controlled site-specific and oriented immobilization can be achieved. As the hexa-histidine tag (His-tag) [113] is one of the most commonly used affinity tags, a wide range of proteins has been immobilized to different surfaces as it also offers the advantage of reversibility once in the presence of imidazole. After deposition of the nickel-chelator nitrilotriacetic acid (Ni–NTA) to gold electrodes, the monomeric oxidase laccase [114] and even complexes such as photosystem II (PSII) could be immobilized in a functional form [115]. Similarly, His-tagged AChE was directly immobilized to nickel nanoparticles to develop a biosensor which detected the insecticide paraoxon even at a 10^−13^ M concentration [116]. By using cobalt instead of nickel, a more stable immobilization of avidins and norovirus proteins on sensors for bio-layer interferometry (BLI) biosensor surface was achieved and was stable even in the presence of 0.7 M imidazole [117]. His-tagged alanine racemase from *Geobacillus stearothermophilus* was immobilized on a silica-coated plate that was modified to contain cobalt ions; it retained its activity and was unaltered after treatments of drying, freezing, or immersion in n-hexane [118]. His-tags also bind to platinum and its deposition, together with graphene on paper, allowed functionalization with His-tagged odor-binding proteins and the assembly of paper electrode-detecting neonicotinoid insecticides [119]. In a different approach, peptides can also be designed or screened for binding specifically to the surface of a selected proteins. Once immobilized, these peptides enable a surface to specifically recognize a target protein. Peptides specifically binding β-galactosidase have been reported to hold the enzyme at the surface while preserving a high specific activity, thermal stability, and guaranteeing a controlled protein orientation [120].

Although larger, affinity proteins offer similar strategies to those of affinity peptides regarding immobilization to sensing surfaces (Figure 3f). By fusing a maltose binding protein to a nitroreductase, electrodes that were previously treated with an electropolymerized film of *N*-(3-pyrrol-1-ylpropyl)-4,4′-bipyridine (PPB) were functionalized [121]. Upon immobilization, the nitroreductase retained a higher degree of activity for the analyte 2,4,6-trinitrotoluene (TNT) when immobilized as fusion construct when compared to simple adsorption, reaching a detection limit of 2 μM for TNT. In addition, the enzyme retained an affinity for the substrate similar to the wild-type, i.e., the fusion construct had an apparent *K*_M_ of 95 μM when on the surface; a similar value of 78 μM was measured for the untreated enzyme in solution with TNT [121]. Hydrophobins were used to drive enzymes to a hydrophobic polystyrene surface. Once fused at the gene level to glutathione-S-transferase (GST), a biosensor for the detection of pesticides molinate and captan was produced [122]. The hydrophobin-assisted immobilization of GST resulted in a higher affinity for the analytes and a higher catalytic activity, e.g., a lower *K*_M_ and an almost double *k*_cat_ [122]. With a focus on the oriented immobilization of antibodies in a single monolayer, hydrophobin was genetically fused to protein A (see below) and used to functionalize graphene, producing a sensing surface that resisted drying and with a detection level in the femtomolar range [123]. The tight binding and self-assembly of hydrophobins was exploited as surface pretreatment to modify the properties of both hydrophilic and hydrophobic surfaces, e.g., mica and polydimethylsiloxane (PDMS), whose wettability was changed and resulted in moderately hydrophilic surface [124]. Gold electrode surfaces can be decorated both through thiol groups and affinity peptides as well as through gold-binding proteins (GBP). GBP was fused to a single-chain antibody for the functionalization of a surface plasmon resonance (SPR)-based biosensor in a single-step process and detected even a 0.14 ng/mL concentration of the hepatitis B virus antigen [105]. Silica and glass surfaces are strongly bonded by the bacterial protein L2 (Si-tag) from *E. coli*; this was fused to both the GFP and luciferase with dissociation constants in the nanomolar range and similar levels of enzymatic activity [125]. In a biomimetic approach, proteins were immobilized to silica using silaffins, proteins involved in the precipitation of silica for skeleton formation in diatoms, which produced nano-to-micro spheres and plates in vitro [126]. A minimal version of silaffins, the peptide R5 (sequence Ser_2_-Lys_2_-Ser-Gly-Ser-Tyr-Ser-(Gly-Ser-Lys)_2_-Arg_2_-Ile-Leu and isoelectric point 11.2) was fused to glucose oxidase that was self-immobilized into silicon dioxide particles; this was later used to monitor the levels of glucose inside eukaryotic cells [127,128]. Interestingly, the R5 peptide also has been proven to precipitate titanium dioxide-forming nanoparticles [129]. The addition of affinity peptides and proteins requires a protein engineering step, unless the conjugation proceeds by chemical means, and provides a controlled site-specific interaction with the surface in an oriented single monolayer. The approach can be widely applied and conditions promoting the detachment from the surface, e.g., for surface regeneration, can be identified as for protein desorption. Because of their small dimensions, affinity peptides can be introduced into proteins of all sizes with minimal interference with the structure and functionality; a result, affinity proteins might be more valuable with proteins of bigger sizes to ensure their maximum exposure on the surface, e.g., avoiding exposure of the underlying layer of surface-bound affinity proteins.

Without requiring a protein engineering step, pre-treatment of surfaces with proteins which offer a specific, tight, and reversible binding such as protein A and streptavidin can be used to achieve an oriented protein immobilization. Protein A from *Staphylococcus aureus* has been widely used for the affinity-based immobilization of immunoglobulins (antibodies) and streptavidin to capture biotin and, accordingly, proteins that have been biotinylated. Protein A is naturally anchored to the bacterial surface; however, it is now commercially available in a recombinant form of approx. 35 kDa that is characterized by the presence of five homologous immunoglobulin-binding sites formed by three anti-parallel α-helices. As the binding to antibodies occurs with dissociation constants in the nanomolar range through the heavy chains, there is no interference with the antigen recognition [130]. Protein A is very stable in a 1–11 pH range, at 6 M guanidine hydrochloride and at 80 °C [131]. However, extensive adsorption of protein A can lead to denaturation and loss of functionality, as reported for the multi-layer arrangement observed on silicon [132]. Conjugated to a wide range of resins used for antibody purification, protein A has also been applied in biosensors. As a result of its binding specificity, protein A has been used to achieve an oriented immobilization of antibodies to surfaces. Immobilization via protein A has been proven to be superior in preserving the affinity of antibodies than chemical crosslinking on various substrates [133] on a fiber optic biosensor [134]; its immobilization to a nanostructured gold surface, e.g., formed by nano-sized gold particles, has proved to retain a higher functionality of the antibodies as compared to a flat gold surface and a glutaraldehyde-based approach [135]. In an affinity-based approach aimed at preserving the conformation, a gold-affinity peptide (sequence Met-His-Gly.Lys-Thr-Gln-Ala-Thr-Ser-Gly-Thr-Ile-Gln-Ser) was fused to a version of protein A with only two antibody-binding domains. An enhanced performance was achieved on SPR sensors as compared to chemical immobilization methods, reaching a detection limit 0.5 mg mL^−1^ for the human growth hormone (hGH) [136]. With regard to potential medical diagnostic applications, protein A also has been fused to a cellulose-binding domain and directly applied to the low-fouling surface of cellulosic microtitre plates [137]. On the other hand, streptavidin is a 52 kDa tetrameric protein produced by streptomycetes which binds non-covalently but with affinity in the femtomolar range, i.e., *K*_d_ ∼10^−14^ M [138,139], four biotin molecules. Proteins to be bound by streptavidin can be biotinylated at their surface-exposed amine groups using various commercially available solutions such as biotin-NHS reagents; however, this produces an unpredictable and heterogeneous modification that might compromise the bioactivity of antibodies more than a site-specific approach [140]. Site-specific biotinylation might be more desirable for an oriented immobilization and can be achieved with in vivo [141,142,143] or in vitro methods using specific peptides as acceptors [144,145], sugar-mediated strategies [67], or inteins [146]. The immobilization of streptavidin has been extended to surfaces not offering the desired chemical groups, namely poly(methyl methacrylate) (PMMA) and polystyrene, by using oxygen plasma to introduce functional groups (e.g. carboxylic groups) to the surface to be later used for immobilization with the EDC chemistry [147,148].

As a concluding remark, the functionalization of sensing surfaces can proceed through various straightforward but less controllable approaches; these include chemical crosslinking and adsorption as well as more selective aiming at a controlled protein orientation on the surface such as by affinity, enzymatic crosslinking, or the use of a protein A-like strategy. A thorough *in silico* and experimental analysis of the surface of the protein to identify pronounced regions of hydrophobicity, polarity, disulfide bonds, or residues susceptible to crosslinking with enzymes can suggest the most efficient immobilization approach. Protein engineering widens the possibilities and the fusion to affinity peptides or proteins provides a direct single-step immobilization process. In all cases, the study of the physico-chemical properties of not only the material surface but also of the protein, with its structural and surface features, is a crucial initial step towards the selection of an immobilization approach that can provide ease of assembly and optimal biosensor performance.
